# Letter from the Editor in Chief

**DOI:** 10.19102/icrm.2023.14106

**Published:** 2023-10-15

**Authors:** Moussa Mansour


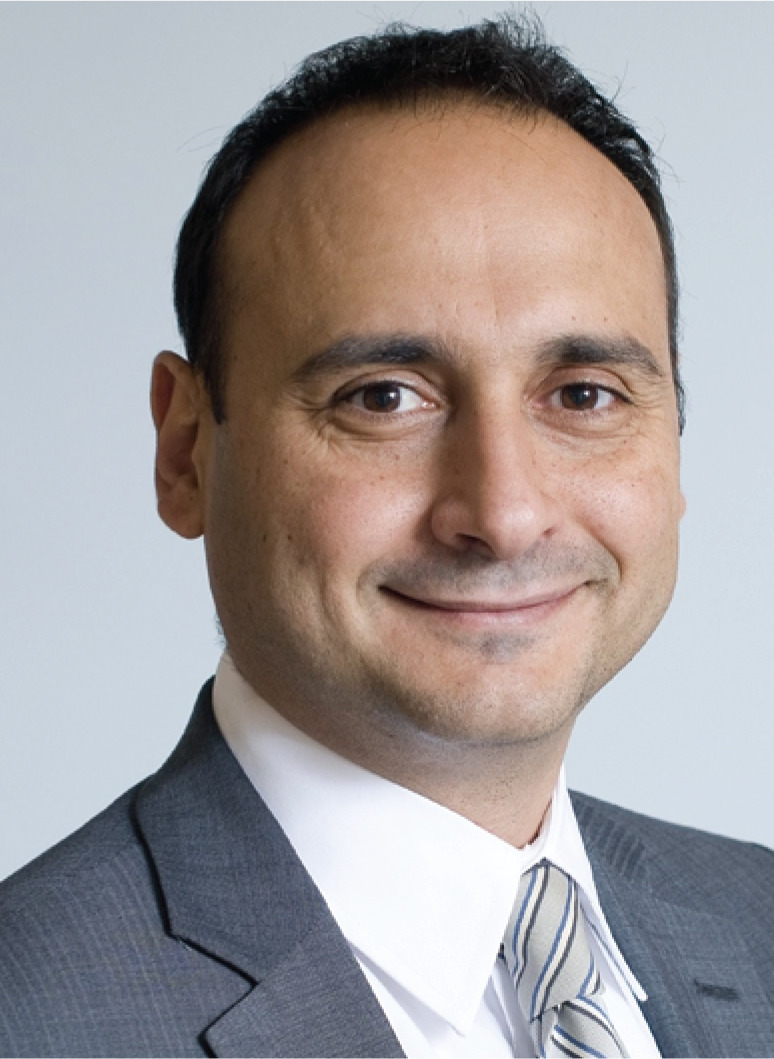


Dear readers,

Interest in physiologic pacing, including left bundle area pacing, has grown over the past few years due to the lack of responsiveness to biventricular pacing in many patients and the deleterious effect of chronic right ventricular (RV) pacing. However, current literature estimates of the burden of RV pacing in patients undergoing pacemaker implantation vary widely.^1,2^ As such, better knowledge of the rate of RV pacing may facilitate the adoption of physiologic pacing over RV pacing.

This issue of *The Journal of Innovations in Cardiac Rhythm Management* contains an important article titled “Exploring the Temporal Patterns of Right Ventricular Pacing Burden,” in which Chattopadhyay et al.^3^ report the findings of a retrospective study analyzing the burden of RV pacing in 1263 patients who underwent pacing for bradycardia during a 5-year period. The study contains several important findings, including that the overall burden of RV was higher than previously reported and remained stable over time. Also, patients with sinus node dysfunction continued to have a low burden of RV pacing when the initial P–R interval was <250 ms, but this was not true in cases where the interval was >250 ms, wherein the burden of RV pacing exceeds 70%.

Biventricular pacing is superior to conventional RV pacing in patients with atrioventricular block and left ventricular (LV) dysfunction.^4^ Meanwhile, there are no data concerning the role of advanced pacing strategies, such as physiologic pacing, in patients with bradycardia and preserved LV function. The described study by Chattopadhyay et al. reporting that all patient groups, except those with sinus node dysfunction and short P–R intervals, can be expected to have a high burden of RV pacing makes a strong case for considering physiologic pacing in most patients, even when the LV function is preserved. At a minimum, the study findings highlight an urgent need for the completion of randomized studies comparing physiologic and RV pacing in patients with normal LV function. This study and others may accelerate the development of technologies to facilitate and improve physiologic pacing.


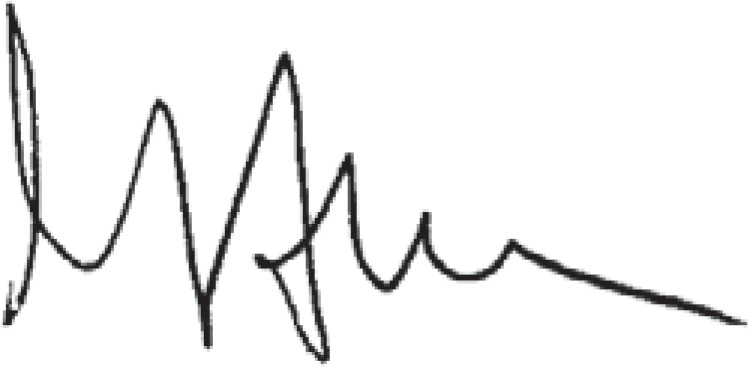


Sincerely,

Moussa Mansour, md, fhrs, facc

Editor in Chief

*The Journal of Innovations in Cardiac Rhythm Management*

MMansour@InnovationsInCRM.com

Director, Atrial Fibrillation Program

Jeremy Ruskin and Dan Starks Endowed Chair in Cardiology

Massachusetts General Hospital

Boston, MA 02114
